# Survival Rate of Gastric Cancer Patients in Jordan: Secondary Data Analysis

**DOI:** 10.2196/14359

**Published:** 2020-05-04

**Authors:** Ashraf Aqel, Yousef Khader, Kamal Arqoub, Omar Nimri

**Affiliations:** 1 Jordan Field Epidemiology Training Program Ministry of Health Amman Jordan; 2 Department of Community Medicine, Public Health and Family Medicine Faculty of Medicine Jordan University of Science and Technology Irbid Jordan; 3 Ministry of Health Amman Jordan

**Keywords:** gastric cancer, survival rate, Jordanian cancer cases

## Abstract

**Background:**

Gastric cancer accounts for 2.7% of all newly diagnosed cancer cases in Jordan.

**Objective:**

The aim of this study was to calculate the survival rate and its determinants among Jordanian patients who were diagnosed with gastric cancer between 2010 and 2014.

**Methods:**

A descriptive study was conducted based on secondary analysis of data from the Jordan Cancer Registry during the period of 2010-2014. Only cancer-related deaths were recorded as “death” in the survival analysis.

**Results:**

A total of 1388 new cases of gastric cancer were recorded between 2010 and 2014. Of these, 872 (62.8%) were Jordanians and 60.5% were males. The mean age at diagnosis was 58.9 years and the median follow-up time was 1.6 years. The 5-year survival rate decreased significantly from 89% in patients with well-differentiated cancer to 32% in patients with poorly differentiated cancer (*P*=.005). The overall 5-year survival rate was 37.7% and the median survival was 1.48 years (95% CI 1.179-1.783). The 5-year survival rate decreased significantly with increasing age and with advanced stage of the disease: the 5-year survival rate was 75% for localized-stage, 48% for regional-stage, and 22.7% for distant-metastasis disease (*P*=.005).

**Conclusions:**

This study showed that the overall 5-year survival rate among patients with gastric cancer in Jordan between 2010 and 2014 was 37.7%, which is higher than the reported rates from different countries in the Eastern Mediterranean region such as Egypt.

## Introduction

Gastric cancer, also known as stomach cancer, develops from the lining layers of the gastrointestinal tract. The cancer may spread from the gastric region to other parts of the body, particularly the liver, lungs, bones, lining of the abdomen, and lymph nodes. The cancer survival rate measures the proportion of people with cancer who will be alive at a certain time after diagnosis, given that they did not die from a cause other than their cancer. Survival rates are important for prognosis, social planning, new intervention evaluation, and future expectation.

Gastric cancer currently ranks fourth in cancer incidence worldwide and is the most common type of cancer among Japanese men [1]. More than 70% of cases occur in developing countries [1]. The gastric cancer incidence rate differs among regions in the Middle East, from very high in Iran (26.1/100,000) to low in Lebanon (6/100,000) and very low in Egypt (3.4/100,000), although all countries are classified as developed at an intermediate socioeconomical level [2-4]. Epidemiological studies show that the prevalence of *Helicobacter pylori* infection is similar in these countries, with a particularly high level of infection in childhood. People who are infected with *H. pylori* are also up to 8 times more likely to develop a certain kind of stomach cancer; however, this bacterium is only one of the possible causes of stomach cancer. Smoking, a diet low in fruit and vegetables, and a history of stomach surgeries can also raise the risk. Nevertheless, *H. pylori* infection prevalence, distribution pattern of virulence factors, diet, and smoking could not adequately explain the observed differences in cancer rates. This reflects the multifactorial etiology of gastric cancer, and suggests that *H. pylori* infection does not always directly correlate with the risk of gastrointestinal diseases such as gastric cancer.

In Jordan, gastric cancer accounts for 2.7% of all newly diagnosed cancer cases, and affects men more frequently than women with a ratio of 1.7:1. Gastric cancer contributes to 4.6% of all deaths due to all types of cancer, ranking sixth among the top 10 cancer-related mortality causes in Jordan [4]. The number of cases of gastric cancer increased in 2010-2014, reaching the ninth position of the top 10 causes of cancer for men in Jordan and the sixth cause of cancer-related deaths. However, very few studies have investigated the survival of gastric cancer and its determinants [5]. Therefore, the aim of this study was to calculate the survival rate and evaluate its determinants among Jordanian patients who were diagnosed with gastric cancer between 2010 and 2014.

## Methods

This study was based on data from the Jordan Cancer Registry, which accounts for more than 95% of all cancer cases in Jordan. The Jordan Cancer Registry uses forms for data collection on sociodemographic characteristics, including national identification number, name, age, marital status, and address, and information related to cancer, including histopathology, morphology, stage of cancer, location of tumor, date of diagnosis, date of last visit, and outcome. All gastric cancer cases among Jordanians who were registered in the Jordan Cancer Registry during the period of 2010-2014, with or without a histopathology report, were included in the study and the data were analyzed using survival analysis. Patients with multiple cancers were not included in this study.

The demographic and clinical characteristics of each registered patient were obtained from the Jordan Cancer Registry files and hospital medical records through the standard data request form. Data on the type and stage of cancer were obtained from histopathology reports from governmental and private laboratories in addition to the medical records of hospitals. The histopathology type was categorized according to the cancer site. The cancer stage was classified into localized, regional, distant metastasis, and unknown stage.

To identify the vital status of these patients, the date of the last visit was obtained from the medical records. In addition, the vital status was ascertained from the Civil Registration Department using a unique national identification number. Only cancer-related deaths were recorded as “death” in the survival analysis. The few noncancer-related deaths, as ascertained from the Civil Registration Department, were considered as censored cases. The period of observation was set for the included patients from the date of diagnosis to the last date of observation if the patient was alive (December 31, 2016) and to the date of death if the patient died during the observation period. The follow-up end point was death from cancer. Ethical approval was obtained from the Institutional Review Board at the Ministry of Health.

Data were analyzed using Statistical Package for Social Sciences Software (SPSS) version 23 (IBM, New York, NY, USA). Data are described using means and percentages. The overall survival was estimated using the Kaplan-Meier product limit technique. The log-rank test was used to compare survival rates between groups. Cox regression analysis was used to determine factors associated with the time to death. *P*<.05 was considered statistically significant.

## Results

A total of 1388 new cases of gastric cancer were recorded during the period of 2010-2014. Of these, 872/1388 (62.8%) were Jordanians, and 60.5% were males and 39.5% were females. The mean age at diagnosis was 58.9 years (59.7 years for males and 57.8 years for females). Almost half of the patients (48.4%) were above 60 years of age. The most commonly affected age group was 60-69 years. The majority of patients were married. Approximately 20.4% were current or past smokers. The grade of the tumor was poorly differentiated in 39.6% of the cases, and 37.9% of cases had an unknown stage. Approximately 40.3% of all cases underwent surgical interventions and 31% had received chemotherapy. The follow-up ranged from 0 to 7.1 years with a mean of 1.5 years. The median follow-up time was 1.6 years.

**Figure 1:**
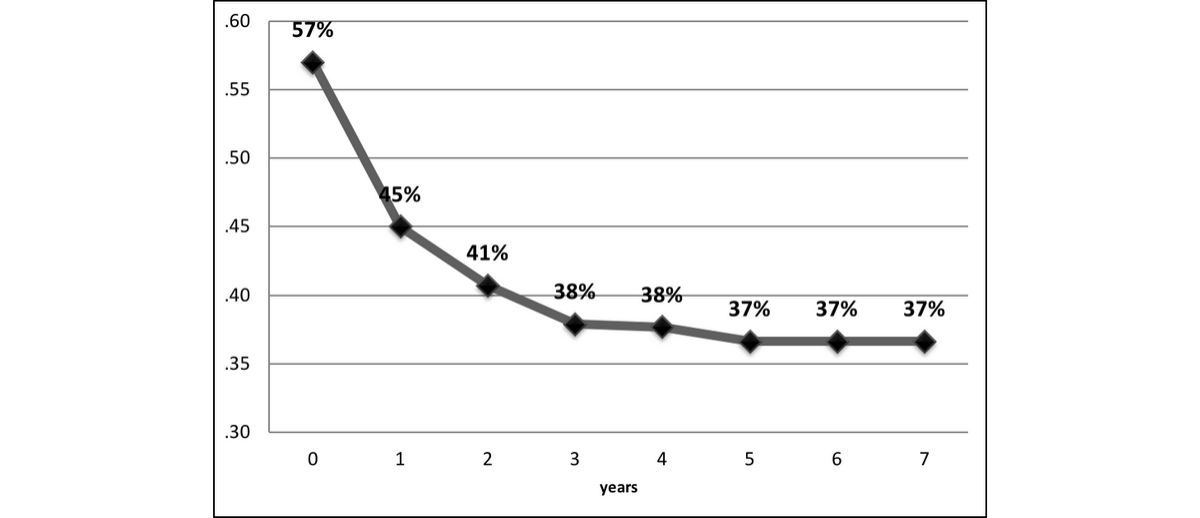
The proportions of Jordanian patients surviving gastric cancer by year.

The proportion of patients surviving at each time interval and the cumulative survival are shown in Figure 1.

The overall 5-year survival rate was 37.7%, and the median survival was 1.48 years (95% CI 1.179-1.783). The 5-year survival rate was 93.5% among non-Jordanian patients and 37.7% among Jordanian patients (*P*=.005). The 5-year survival rate was 89% in patients with well-differentiated cancer and 32% in patients with poorly differentiated cancer (*P*=.005). The 5-year survival rate decreased significantly according to age group, from 43% for patients <40 years old to 29.8% for patients ≥70 years old (*P*=.005). The survival rate also decreased significantly with advanced stage of the disease: the 5-year survival rate was 75% for localized stage, 48% for regional stage, and 22.7% for distant metastasis (*P*=.005). The median survival for patients with gastric cancer according to demographic and clinical characteristics is summarized in Table 1. The 5-year survival rate of patients receiving surgical procedures with neither chemical nor radiological therapy was 43.3%. Patients who were treated with chemotherapy only had a 5-year survival rate of 27.8%, and patients who received radiotherapy only had a 5-year survival rate of 15.7%. The 5-year survival rate was 43.1% for patients who did not undergo any therapy.

Table 2 shows the results of the multivariate analysis of factors associated with the hazard of death from Cox regression analysis. The only factors that were significantly associated with death were age, nationality, and grade and stage of cancer. The hazard ratio (HR) of death increased significantly with increased age, and was the highest for the group aged ≥70 years (HR=1.68). The hazard of death increased significantly among Jordanian patients compared to non-Jordanian patients (HR=5.27). The hazard of death was significantly higher for those with poorly differentiated cancer compared to those with well-differentiated cancer (HR=5.93). The hazard was also much higher for patients whose cancer stage was regional (HR=2.35) and in those with distant metastasis (HR=5.65) compared to those with localized cancer.

**Table 1:** Median survival time for patients with gastric cancer according to demographic and clinical characteristics.

Patient characteristics		Median survival time			*P* value
		Estimate	SE	95% CI	
**Gender**					.75
	Male	2.12	0.29	1.54-2.70	
	Female	2.17	0.90	0.40-3.94	
**Age (years)**					<.001
	<40	2.11	0.80	0.52-3.70	
	40-49	—	—	—	
	50-59	3.42	N/A^a^	N/A	
	60-69	2.47	0.78	0.94-4.00	
	≥70	1.06	0.20	0.67-1.46	
**Marital status**					.69
	Single	2.28	1.62	0.00-5.47	
	Married	2.12	0.27	1.59-2.65	
**Smoking status**					<.001
	Never smoked	1.80	0.28	1.26-2.35	
	Current smoker	1.47	0.26	0.96-1.97	
	Past smoker	1.11	0.62	0.00-2.32	
**Site**					.20
	Cardia, NOS^b^	1.84	0.33	1.20-2.49	
	Fundus of stomach	5.64	2.58	0.58-10.70	
	Body of stomach	2.46	0.48	1.51-3.41	
	Lesser curvature of stomach	2.01	1.17	0.00-4.30	
	Overl. lesion of stomach	0.95	0.40	0.17-1.74	
	Stomach, NOS	2.03	0.38	1.27-2.78	
**Grade**					<.001
	Well-differentiated	—	—	—	
	Moderately differentiated	1.98	0.35	1.30-2.66	
	Poorly differentiated	1.26	0.13	1.00-1.52	
	Undifferentiated/Anaplastic	1.87	0.93	0.03-3.71	
	Unspecified	2.47	0.98	0.55-4.39	
**Stage**					<.001
	Localized	—	—	—	
	Regional direct extend	2.96	2.15	0.00-7.18	
	Regional direct extend and lymph node stage 2	1.88	0.35	1.18-2.58	
	Distant stage 3 and 4	0.89	0.09	0.70-1.07	

^a^N/A: not applicable.

^b^NOS: not otherwise specified.

**Table 2:** Multivariate analysis of factors associated with the hazard of death in Cox regression analysis.

Category		Hazard ratio		95% CI		*P* value		
**Age (years)**								
	≤40		1.00		—		—	
	40-49		0.88		0.64-1.21		.44	
	50-59		0.80		0.59-1.10		.18	
	60-69		0.96		0.71-1.29		.78	
	≥70		1.68		1.26-2.24			<.001
**Nationality**								
	Non-Jordanian		1.00		—			—
	Jordanian		5.26		3.58-7.73			<.001
**Grade**								
	Well-differentiated		1.00		—			—
	Moderately differentiated		4.74		1.74-12.95			.002
	Poorly differentiated		5.93		2.20-16.00			<.001
	Undifferentiated/Anaplastic		5.93		1.58-22.31			.008
	B cell		2.08		0.74-5.82			.16
	Unspecified		4.91		1.81-13.31			.002
**Summary stage**								
	Localized		1.00		—			—
	Regional direct extend		2.35		1.34-4.12			.003
	Regional lymph node		1.50		0.79-2.86			.21
	Regional direct extend and lymph node		3.07		1.83-5.14			<.001
	Regional NOS^a^ Stage 2		4.08		2.06-8.09			<.001
	Distant Stage 3&4		5.65		3.52-9.08			<.001
	Unknown		2.90		1.80-4.65			<.001

^a^NOS: not otherwise specified.

## Discussion

Data on the survival analysis of gastric cancer in Eastern Mediterranean countries are scarce, including Jordan. Previous studies in other countries have reported variable gastric cancer survival rates. Approximately 71% of gastric cancer cases occur in less developed countries, with the highest incidence reported in Asia, Latin America, and the Caribbean, and the lowest incidence in Africa and North America [6]. The Republic of Korea was reported to have the highest rate of gastric cancer, followed by Mongolia and Japan [6]. A case-control study indicated that several food items and cooking methods were associated with an increased or decreased risk of stomach cancer among Koreans [2]. Specifically, an increased risk of stomach cancer was noted among people who frequently consumed broiled meats and fishes, salted side dishes (salted/fermented fish products), and salty stewed foods such as soybean paste thick stew. Frequent consumption of mango bean pancake, tofu, cabbage, spinach, and sesame oil decreased the risk. Analysis by cooking method showed that the risk of stomach cancer from the same foods varied according to the preparation method. For meat and fish, pan frying was associated with a decreased risk, whereas stewing or broiling was associated with an increased risk. This study showed that the overall 5-year survival rate was 37.7% for all patients in Jordan, with an estimated median of 1.481 years (95% CI 1.179-1.783). This rate is higher than those reported from different countries in the Eastern Mediterranean region, including Egypt with a median overall survival rate of 6 months (95% CI 3.3-8.9) [7].

Various studies from Iran have reported a 5-year survival rate of gastric cancer of 12.8% [5]. The disparities in gastric cancer survival among Eastern Mediterranean countries may be attributed to several factors, including differences in socioeconomic status, stage at diagnosis, treatment, physician characteristics, and hospital factors. The better survival in Jordan compared with other countries in the region might be explained by the fact that cancer care in Jordan is more advanced in comparison to that of most neighboring countries, and the country hosts many local and Western-trained physicians who can deliver various cancer treatment modalities [8]. Currently, the King Hussein Cancer Foundation and Center (KHCC) treats around 60% of all cancer cases in Jordan. The KHCC is a specialized tertiary-care hospital that provides all treatment modalities and services to Jordanian patients as well as other patients from neighboring countries. However, further studies are needed to examine the differences in gastric cancer survival between these countries. There was no significant difference in the survival rate between men and women in the univariate analysis and multivariate analysis. This lack of gender difference in survival rate was also reported in some of the previous studies mentioned above.

This study showed that the hazard of death increased significantly with increased age, and the highest hazard was found in the age group ≥70 years. This result was similar to previous studies [5,7] showing that older patients had a poorer survival rate compared to younger patients. The contradictory results of previous studies on age may be due to inclusion of patients from single referral centers and poor adjustment for the effect of possible confounders.

Different clinical and pathological prognostic factors have been proposed for gastric cancer in the literature to date, including location of the tumor, tumor stage, differentiation of the tumor, and surgical and distant metastasis. The present multivariate analysis using Cox regression showed that age, nationality, grade, and stage were significant predictors of survival. The hazard of death was significantly higher for patients aged >70 years compared to those in other age categories due to the increased probability of death with increasing age. The higher hazard of death for Jordanian patients compared to non-Jordanian patients may be explained by the shorter period of follow-up for non-Jordanian patients because they departed after they received medical treatment and therefore could not be followed up. The hazard of death was also significantly higher for those with poorly differentiated cancer compared to those with well-differentiated cancer. Moreover, it was much higher for patients whose cancer stage was regional and those with distant metastasis compared to those with localized cancer. Therefore, the earlier the stage at diagnosis, the higher the chance of survival. The differences in survival according to stage are explained by the differences in the extent to which the cancer has spread and how many lymph nodes have been affected.

Data from the Jordan Cancer Registry should be interpreted with caution. Similar to many registries in the region, the Jordan Cancer Registry does not collect information on other possible predictors of mortality such as occupation, level of education, economic status, and comorbidity. Therefore, our HR estimates might be biased owing to the lack of adjustment for the effect of unmeasured variables.

In conclusion, this study showed that the overall 5-year survival rate among patients with gastric cancer in Jordan was 37.7%, which is higher than the reported rates from different countries in the Eastern Mediterranean region, including Egypt. Increased age, poor differentiation, and advanced cancer stage were associated with lower survival rates. The survival rate of patients who underwent surgical interventions alone was 43.3%, whereas that among patients who received chemotherapy or radiotherapy alone was 27.8% and 15.7%, respectively, which differs from the results of other regional studies. This finding may be explained by the fact that patients underwent surgical interventions at an early stage of cancer, whereas chemotherapy and radiotherapy are given to patients with much worse cases. It is well established that gastric cancer progression can be largely prevented by early detection and removal of the adenomatous tissues, and survival is therefore significantly better when gastric cancer is diagnosed while still localized. Therefore, improved screening strategies are needed for the early detection of gastric cancer.

## Abbreviations

HR: hazard ratio
KHCC: King Hussein Cancer Foundation and Center
NOS: not otherwise specified
